# Supplementary material for: Modeling glioblastoma heterogeneity as a dynamic network of cell states
Source: Mol Syst Biol. 2021 Sep 16;17(9):e10105. doi: 10.15252/msb.202010105 (PMC8444284; doi:10.15252/msb.202010105)
Supplement: Supplementary file 5 — Source Data for Figure 3 [file MSB-17-e10105-s001.zip › Figure3A_sourcedata/GSEA_3065/hallmarks_state4.GseaPreranked.1623416296068/HALLMARK_MYC_TARGETS_V2.html]

Details for gene set HALLMARK\_MYC\_TARGETS\_V2[GSEA]

|  || Dataset | state4 |
| Phenotype | NoPhenotypeAvailable |
| Upregulated in class | na\_neg |
| GeneSet | HALLMARK\_MYC\_TARGETS\_V2 |
| Enrichment Score (ES) | -0.6832044 |
| Normalized Enrichment Score (NES) | -2.0132651 |
| Nominal p-value | 0.0 |
| FDR q-value | 0.0 |
| FWER p-Value | 0.0 |
Table: GSEA Results Summary

  

Fig 1: Enrichment plot: HALLMARK\_MYC\_TARGETS\_V2      
 Profile of the Running ES Score & Positions of GeneSet Members on the Rank Ordered List

  

| PROBE | GENE SYMBOL | GENE\_TITLE | RANK IN GENE LIST | RANK METRIC SCORE | RUNNING ES | CORE ENRICHMENT || 1 | SRM |  |  | 2657 | 0.032 | -0.4168 | No |
| 2 | NDUFAF4 |  |  | 2882 | 0.027 | -0.4447 | No |
| 3 | CDK4 |  |  | 3394 | 0.016 | -0.5220 | No |
| 4 | PES1 |  |  | 3662 | 0.007 | -0.5628 | No |
| 5 | NOP16 |  |  | 3977 | -0.008 | -0.6108 | No |
| 6 | WDR74 |  |  | 3997 | -0.010 | -0.6110 | No |
| 7 | EXOSC5 |  |  | 4150 | -0.018 | -0.6299 | No |
| 8 | NOC4L |  |  | 4181 | -0.021 | -0.6285 | No |
| 9 | BYSL |  |  | 4357 | -0.032 | -0.6470 | No |
| 10 | PHB |  |  | 4584 | -0.050 | -0.6683 | Yes |
| 11 | MYC |  |  | 4618 | -0.053 | -0.6580 | Yes |
| 12 | GNL3 |  |  | 4704 | -0.060 | -0.6537 | Yes |
| 13 | HSPE1 |  |  | 4744 | -0.063 | -0.6411 | Yes |
| 14 | PPAN |  |  | 4789 | -0.067 | -0.6282 | Yes |
| 15 | NIP7 |  |  | 4881 | -0.074 | -0.6209 | Yes |
| 16 | SLC19A1 |  |  | 5083 | -0.090 | -0.6263 | Yes |
| 17 | UNG |  |  | 5148 | -0.096 | -0.6078 | Yes |
| 18 | DDX18 |  |  | 5207 | -0.102 | -0.5868 | Yes |
| 19 | PLK4 |  |  | 5210 | -0.102 | -0.5567 | Yes |
| 20 | CBX3 |  |  | 5377 | -0.116 | -0.5487 | Yes |
| 21 | DCTPP1 |  |  | 5588 | -0.143 | -0.5398 | Yes |
| 22 | MRTO4 |  |  | 5738 | -0.169 | -0.5133 | Yes |
| 23 | PLK1 |  |  | 5836 | -0.192 | -0.4717 | Yes |
| 24 | PA2G4 |  |  | 5841 | -0.193 | -0.4149 | Yes |
| 25 | NPM1 |  |  | 5884 | -0.203 | -0.3612 | Yes |
| 26 | NOP56 |  |  | 5921 | -0.213 | -0.3036 | Yes |
| 27 | HSPD1 |  |  | 5967 | -0.227 | -0.2432 | Yes |
| 28 | MCM5 |  |  | 6069 | -0.278 | -0.1765 | Yes |
| 29 | TMEM97 |  |  | 6131 | -0.324 | -0.0899 | Yes |
| 30 | MCM4 |  |  | 6171 | -0.372 | 0.0148 | Yes |
Table: GSEA details [plain text format]

  

Fig 2: HALLMARK\_MYC\_TARGETS\_V2: Random ES distribution      
 Gene set null distribution of ES for **HALLMARK\_MYC\_TARGETS\_V2**
